# Supplementary figures and images for: Association between methylmalonic acid and Alpha-Klotho in American adults: A cross-sectional study
Source: PLoS One. 2025 Dec 30;20(12):e0337285. doi: 10.1371/journal.pone.0337285 (PMC12752970; doi:10.1371/journal.pone.0337285)

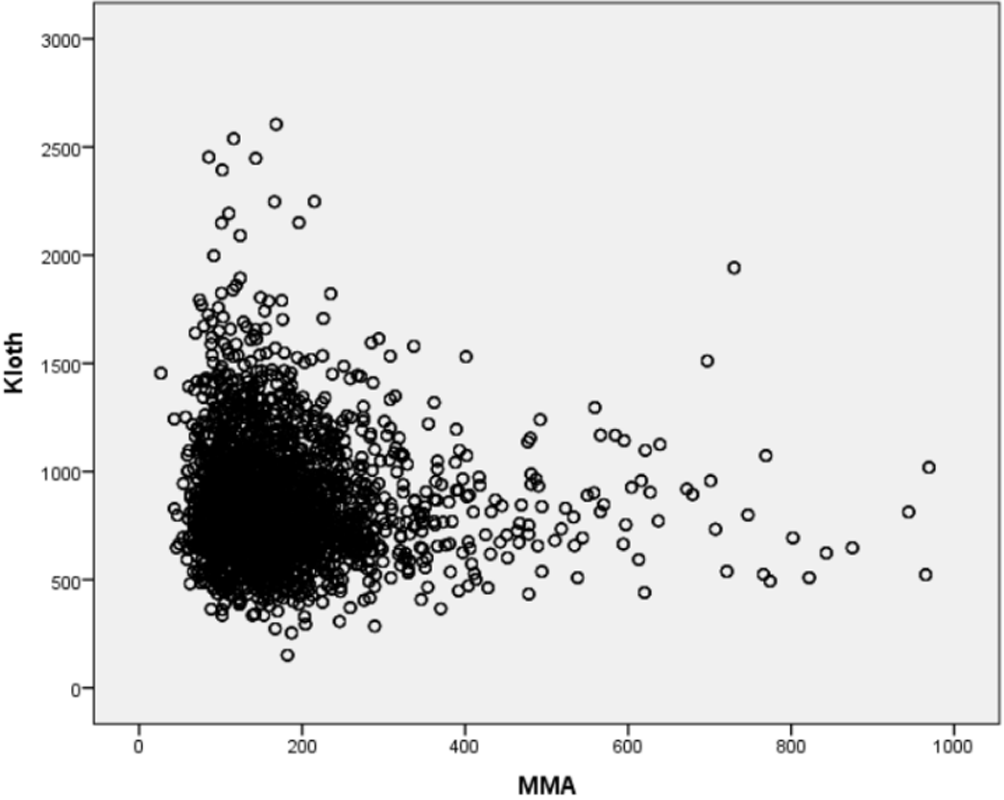

Supplement: S1 Fig — (PNG) [file pone.0337285.s001.png]
