# Supplementary material for: Association between methylmalonic acid and Alpha-Klotho in American adults: A cross-sectional study
Source: PLoS One. 2025 Dec 30;20(12):e0337285. doi: 10.1371/journal.pone.0337285 (PMC12752970; doi:10.1371/journal.pone.0337285)
Supplement: S2 File — (DOCX) [file pone.0337285.s003.docx]

README

The provided data is for people aged 20 and above

Variable assignment encoding

1. **Sex**

1-male

2-female

1. **Race**

1-Non-Hispanic White

2-Non-Hispanic Black

3-Mexican American

4-Other Hispanic

5-Other Race - Including Multi-Racial

1. **Marry**

1-Married/ Living with partner

2-Never married/Other: widowed, divorced, or separated individuals

1. **Education**

1-Less than high school: Less Than 9th Grade and 9-11th Grade (Includes 12th grade with no diploma)

2-High school or equivalent: High school graduate/GED or equivalent

3-Above high school: Some College or AA degree and College graduate or above

1. **Age_group.40.60**

1-<40

2-40-59

3-≥60

1. **PIR_group1**

1-≤1.30

2-1.31-3.50

3->3.50

1. **smoke**

1-never

2-former

3-now

1. **drink**

1-never

2-former

3-current

1. **CVD**

0-no

1-yes

1. **coronary.heart.disease**

0-no

1-yes

1. **congestive.heart.failure**

0-no

1-yes

1. **Hypertension**

0-no

1-yes

1. **Pregnant**

0-no

1-yes

2-NA

1. **Hyperlipidemia**

0-no

1-yes

**变量定义**

1. **hei2015_total_score**

健康饮食指数-2015，具体算法见参考文献

参考文献：Update of the Healthy Eating Index: HEI-2015

1. **smoke**

吸烟状态，定义如下

never: smoked less than 100 cigarettes in life

former: smoked more than 100 cigarettes in life and smoke not at all now

now: smoked moth than 100 cigarettes in life and smoke some days or every day

参考文献：Association of Dietary Live Microbes and Nondietary Prebiotic/Probiotic Intake With Cognitive Function in Older Adults: Evidence From NHANES

1. **drink1和drink2（饮酒定义）**

饮酒状态，定义如下，345为现在饮酒的不同程度，可以把它们合并为一个current

**1999-2018**

（1）never: had <12 drinks in lifetime

（2）former: had ≥12 drinks in 1 year and did not drink last year, or did not drink last year but drank ≥12 drinks in lifetime

（3）current heavy alcohol use: ≥3 drinks per day for females, ≥4 drinks per day for males, or binge drinking [≥4 drinks on same occasion for females, ≥5 drinks on same occasion for males] on 5 or more days per month)

（4）current moderate alcohol use: ≥2 drinks per day for females, ≥3 drinks per day for males, or binge drinking ≥2 days per month)

（5）current mild alcohol use: ≤1 drinks per day for females, ≤2 drinks per day for males

参考文献：Inverse Association of Telomere Length With Liver Disease and Mortality in the US Population

**2017-2020**

1. never：一生中，没喝过一次任何种类的酒
2. former：一生中有喝但上一年没有喝
3. mild：同上
4. moderate：同上
5. heavy：同上
6. **PA_total_time**

每周运动的时间，具体包括以下五个项目

1. walk or bicycle
2. task around home or yard
3. muscle strength（仅1999-2006）
4. work activity
5. recreational activity

参考文献：

Association of Dietary Live Microbes and Nondietary Prebiotic/Probiotic Intake With Cognitive Function in Older Adults: Evidence From NHANES

Political Orientation and Public Attributions for the Causes and Solutions of Physical Inactivity in Canada: Implications for Policy Support

1. **PA_total_time_all0**

由于计算逻辑，对于变量PA_total_time，若本周没有运动（即运动时间为0），此人变量的值为NA，会导致缺失较多，变量PA_total_time_all0将没有运动的人从新定义为0，以保留样本量

1. **PA_total_MET**

将时间转化为活动当量

MET values vary with the type of exercise, and the NHANES offers the recommended MET values for each PA. PA was based on the MET values of type, frequency, and duration of activities per week, which was calculated using the following formula: PA (MET-min/wk) = MET × weekly frequency × duration of each PA.

参考文献：

Association Between Joint Physical Activity and Dietary Quality and Lower Risk of Depression Symptoms in US Adults: Cross-sectional NHANES Study

Risk/benefit tradeoff of habitual physical activity and air pollution on chronic pulmonary obstructive disease: findings from a large prospective cohort study.

1. **PA_total_MET_all0**

同PA_total_time_all0

1. **CVD**

心血管疾病病史，定义如下：

自我报告既往被诊断为冠心病（coronary heart disease）、心绞痛（angina）、卒中（stroke）、心肌梗死（heart attack）、心衰（congestive heart failure），满足其一即可诊断

1. **coronary.heart.diseas**

**angina**

**stroke**

**heart attack**

**congestive heart failure**

均为自我报告

1. **Hypertension**

高血压，诊断如下，满足其一即可：

average blood presure was calculated by the following protocol:

（1）The diastolic reading with zero is not used to calculate the diastolic average.

（2）If all diastolic readings were zero, then the average would be zero.

（3）If only one blood pressure reading was obtained, that reading is the average.

（4）If there is more than one blood pressure reading, the first reading is always excluded from the average.

参考文献：Association of Dietary Live Microbes and Nondietary Prebiotic/Probiotic Intake With Cognitive Function in Older Adults: Evidence From NHANES

**11. DM**

糖尿病，诊断如下，满足其一即可：

The diagnostic criteria for diabetes are:

（1）doctor told you have diabetes,

（2）glycohemoglobin HbA1c(%) >= 6.5,

（3）fasting glucose (mmol/l) >= 7.0,

（4）random blood glucose (mmol/l) >= 11.1,

（5）two-hour OGTT blood glucose (mmol/l) >= 11.1,

（6）Use of diabetes medication or insulin

参考文献：Association of Dietary Live Microbes and Nondietary Prebiotic/Probiotic Intake With Cognitive Function in Older Adults: Evidence From NHANES

**12. Pregnant**

是否怀孕，自我报告

一般需要排除怀孕的参与者，但是回答这个问卷的人比较少，一般不对此问卷纳排，就是说若参与者没有回答这个问题，默认她没怀孕

**13.Hyperlipidemia**

高脂血症的定义，满足任意一条：1)使用降血脂药物；2)高甘油三酯≥150mg/dl；3)高胆固醇（总胆固醇≥200mg/dl，或者LDL≥130mg/dl或者HDL<40mg/dl）

**14.权重变量，加权分析时需要，非加权分析可以忽略**

sdmvpsu

sdmvstra

wtmec2yr

wtmec4yr
