# Supplementary material for: Association between methylmalonic acid and Alpha-Klotho in American adults: A cross-sectional study
Source: PLoS One. 2025 Dec 30;20(12):e0337285. doi: 10.1371/journal.pone.0337285 (PMC12752970; doi:10.1371/journal.pone.0337285)
Supplement: Attachment — Submitted filename: Response to reviewers1.docx [file pone.0337285.s004.docx]

Response to Reviewers

PLOS ONE：PONE-D-25-04628

Association between methylmalonic acid and Alpha-Klotho in American adults: a cross-sectional study

Journal requirements:

When submitting your revision, we need you to address these additional requirements.

1. Please ensure that your manuscript meets PLOS ONE's style requirements, including those for file naming. The PLOS ONE style templates can be found at

https://journals.plos.org/plosone/s/file?id=wjVg/PLOSOne_formatting_sample_main_body.pdf

https://journals.plos.org/plosone/s/file? id=wjVg/PLOSOne_formatting_sample_main_body.pdf<https://journals.plos.org/plosone/s/file?id=ba62/PLOSOne_formatting_sample_title_authors_affiliations.pdf>

**Response to reviewers**

Concern #1: Ensure that the manuscript meets PLOS ONE's style requirements, including those for file naming.

Author response: We have reviewed the manuscript thoroughly and ensured that it adheres to PLOS ONE's style requirements. We utilized the provided PLOS ONE style templates for formatting the main body, title, authors, and affiliations. All file naming conventions have been followed as per the guidelines outlined in the links provided.

2. We noticed you have some minor occurrence of overlapping text with the following previous publication(s), which needs to be addressed:

- https://doi.org/10.3389/fmed.2023.1269671

- https://doi.org/10.1016/j.scitotenv.2022.156938

In your revision ensure you cite all your sources (including your own works), and quote or rephrase any duplicated text outside the methods section. Further consideration is dependent on these concerns being addressed.

**Response to reviewers**

Concern #1: Minor occurrence of overlapping text with previous publications needs to be addressed.

Author response: We have thoroughly reviewed the manuscript and identified the overlapping text with the specified publications. We have rephrased the duplicated text outside the methods section and ensured proper citations for all sources, including our own works. The revised manuscript reflects these changes.

Concern #2: Ensure all sources are cited appropriately.

Author response: We have updated the manuscript to include all necessary citations for our previous works and other relevant sources. Each source is now properly referenced in accordance with the journal's guidelines.

3. Thank you for stating the following financial disclosure:

“Medical Health Research Project of Science and Technology Bureau of Yichang City, Hubei Province (A23-1-021).”

Please state what role the funders took in the study.  If the funders had no role, please state: "The funders had no role in study design, data collection and analysis, decision to publish, or preparation of the manuscript."

If this statement is not correct you must amend it as needed.

Please include this amended Role of Funder statement in your cover letter; we will change the online submission form on your behalf.

**Response to reviewers**

Concern #1: Please state what role the funders took in the study. If the funders had no role, please state: "The funders had no role in study design, data collection and analysis, decision to publish, or preparation of the manuscript."

Author response:The funders also provide certain guidance in the selection of research directions.This amended Role of Funder statement has been included in the cover letter as requested.

4. Your ethics statement should only appear in the Methods section of your manuscript. If your ethics statement is written in any section besides the Methods, please delete it from any other section.

**Response to reviewers**

Concern #1: Your ethics statement should only appear in the Methods section of your manuscript. If your ethics statement is written in any section besides the Methods, please delete it from any other section.

Author response: We have revised the manuscript to ensure that the ethics statement appears only in the Methods section.Any instances of the ethics statement in other sections have been removed accordingly.

5. Please upload a copy of Figure 2, to which you refer in your text on page 4. If the figure is no longer to be included as part of the submission please remove all reference to it within the text.

6. Please include your tables as part of your main manuscript and remove the individual files. Please note that supplementary tables (should remain/ be uploaded) as separate "supporting information" files

**Response to reviewers**

Concern #1:Please upload a copy of Figure 2, to which you refer in your text on page 4. If the figure is no longer to be included as part of the submission please remove all reference to it within the text.

Author response: We have uploaded a copy of Figure 2 as requested. All references to this figure in the text have been retained, as the figure is included in the submission.

Concern #2: Please include your tables as part of your main manuscript and remove the individual files.Please note that supplementary tables (should remain/ be uploaded) as separate "supporting information" files.

Author response:We have incorporated all tables into the main manuscript and removed the individual files as per your instructions.Supplementary tables have been uploaded as separate "supporting information" files.


[Note: HTML markup is below. Please do not edit.]
Reviewers' comments:  
Reviewer's Responses to Questions
**Comments to the Author**  
1. Is the manuscript technically sound, and do the data support the conclusions?
The manuscript must describe a technically sound piece of scientific research with data that supports the conclusions. Experiments must have been conducted rigorously, with appropriate controls, replication, and sample sizes. The conclusions must be drawn appropriately based on the data presented.
Reviewer #1: No

Reviewer #2: Yes

2. Has the statistical analysis been performed appropriately and rigorously?
Reviewer #1: Yes

Reviewer #2: Yes

3. Have the authors made all data underlying the findings in their manuscript fully available?

The [PLOS Data policy](http://track.editorialmanager.com/CL0/http://www.plosone.org/static/policies.action%23sharing/1/010f01956412a40f-56259746-2fce-4df9-bfad-b818cad4c282-000000/fZz_7EaYhJkjW3-0Dc0IGNjtnCZVGagtO0aKaK6lxXs=200) requires authors to make all data underlying the findings described in their manuscript fully available without restriction, with rare exception (please refer to the Data Availability Statement in the manuscript PDF file). The data should be provided as part of the manuscript or its supporting information, or deposited to a public repository. For example, in addition to summary statistics, the data points behind means, medians and variance measures should be available. If there are restrictions on publicly sharing data—e.g. participant privacy or use of data from a third party—those must be specified.
Reviewer #1: No

Reviewer #2: Yes

4. Is the manuscript presented in an intelligible fashion and written in standard English?

PLOS ONE does not copyedit accepted manuscripts, so the language in submitted articles must be clear, correct, and unambiguous. Any typographical or grammatical errors should be corrected at revision, so please note any specific errors here.
Reviewer #1: No

Reviewer #2: Yes

5. Review Comments to the Author
Please use the space provided to explain your answers to the questions above. You may also include additional comments for the author, including concerns about dual publication, research ethics, or publication ethics. (Please upload your review as an attachment if it exceeds 20,000 characters)

**Reviewer #1: Major Issues for Revision
1. Fundamental Conceptual Flaws and Oversimplifications**
The study claims that a negative association between MMA and α-Klotho suggests mitochondrial dysfunction's role in aging. However, correlation does not imply causation, especially in a cross-sectional study. The entire premise that MMA directly reflects mitochondrial dysfunction is questionable.
MMA accumulation is more commonly linked to vitamin B12 deficiency and renal impairment rather than mitochondrial dysfunction.
No direct mechanistic link between MMA and α-Klotho is explored—only speculation.
The authors cite literature suggesting MMA as a mitochondrial dysfunction marker, but many of these studies are context-specific (e.g., genetic disorders, severe metabolic conditions) and cannot be extrapolated to the general population.
→ Solution: The authors need to discuss alternative explanations for their findings (e.g., renal impairment, metabolic dysfunction) rather than assuming mitochondrial dysfunction.

**Response to reviewers**

Concern #1: The study claims that a negative association between MMA and α-Klotho suggests mitochondrial dysfunction's role in aging, but correlation does not imply causation, and the premise that MMA directly reflects mitochondrial dysfunction is questionable.

Author response: We appreciate the reviewer’s concern regarding the interpretation of our findings. In our revised manuscript, we have clarified the limitations of our cross-sectional study design and emphasized that while we observed a negative association between MMA and α-Klotho, this does not establish causation.We have included a discussion on the potential confounding factors, including vitamin B12 deficiency and renal impairment, which may also contribute to MMA accumulation.

Concern #2: MMA accumulation is more commonly linked to vitamin B12 deficiency and renal impairment rather than mitochondrial dysfunction.

Author response: We acknowledge this point and have expanded our discussion to include the established links between MMA, vitamin B12 deficiency, and renal impairment. We have added references to relevant literature that outline these associations and how they may intersect with our findings.

Concern #3:No direct mechanistic link between MMA and α-Klotho is explored—only speculation.

Author response:We agree that our original manuscript did not adequately address the mechanistic pathways linking MMA and α-Klotho. In the revised version, we have included a more detailed exploration of potential mechanisms, supported by recent studies, while also noting the speculative nature of these connections.

Concern #4: The authors cite literature suggesting MMA as a mitochondrial dysfunction marker, but many of these studies are context-specific and cannot be extrapolated to the general population.

Author response: We appreciate this insight and have revised our literature review to highlight the context-specific nature of the studies we cited.We have also emphasized the need for caution when generalizing these findings to the broader population and have included a discussion on the limitations of existing studies.

Concern #5: The authors need to discuss alternative explanations for their findings rather than assuming mitochondrial dysfunction.

Author response: In response to this concern, we have added a comprehensive section discussing alternative explanations for our findings, including renal impairment and metabolic dysfunction. This addition aims to provide a more balanced view of the potential factors influencing MMA levels and their relationship with α-Klotho.

**2. Weakness in Methodology and Study Design**

Cross-sectional design: This prevents causal inference but is not acknowledged adequately.The manuscript implies a directional effect (MMA → α-Klotho decrease), which is misleading.

Many references in the discussion assume causality without direct mechanistic validation.

Longitudinal or interventional studies are needed to confirm such claims.

Serum MMA measurements:

The study relies entirely on NHANES data but does not provide enough quality control details for MMA measurements.

MMA levels fluctuate due to dietary factors, renal function, and transient metabolic changes, which are not sufficiently controlled.

The authors state that MMA was measured by LC-MS/MS, but no intra- or inter-assay variation data are reported.

α-Klotho measurements:

The ELISA method is reported, but batch effects and assay standardization issues are not discussed.

Why were data from 2011–2014 used exclusively when NHANES has additional datasets? This could lead to selection bias.

The reference range used (285.8–1638.6 pg/mL) is outdated and may not reflect inter-laboratory variations.

→ Solution: Acknowledge cross-sectional study limitations, discuss alternative explanations (e.g., renal dysfunction), and provide detailed quality control measures for biochemical assays.

**Response to reviewers**

Concern #1: Cross-sectional design: This prevents causal inference but is not acknowledged adequately. The manuscript implies a directional effect (MMA → α-Klotho decrease), which is misleading.

Author response: We acknowledge the limitations of the cross-sectional design in our study and have added a discussion in the manuscript highlighting this point. We clarify that while our findings suggest an association, they do not imply causation. We have revised the language throughout the manuscript to ensure it does not imply a directional effect without mechanistic validation.

Concern #2: Many references in the discussion assume causality without direct mechanistic validation.

Author response: We have reviewed the references cited in the discussion and have removed or revised those that imply causality without sufficient mechanistic support. We emphasize the need for further research, including longitudinal and interventional studies, to validate the observed associations.

Concern #3: The study relies entirely on NHANES data but does not provide enough quality control details for MMA measurements.

Author response: We have added a section detailing the quality control measures for MMA measurements as provided by NHANES. This includes information on the laboratory protocols and the standards used to ensure the reliability of the data.

Concern #4: MMA levels fluctuate due to dietary factors, renal function, and transient metabolic changes, which are not sufficiently controlled.

Author response: We have acknowledged the potential confounding factors that may influence MMA levels in our revised manuscript. We discuss the implications of these fluctuations and suggest that future studies should consider these variables when interpreting MMA data.

Concern #5: The authors state that MMA was measured by LC-MS/MS, but no intra- or inter-assay variation data are reported.

Author response: We have included information regarding the intra- and inter-assay variation for MMA measurements obtained via LC-MS/MS in the revised manuscript. This data supports the reliability of the measurements used in our study.

Concern #6: The ELISA method is reported, but batch effects and assay standardization issues are not discussed.

Author response: We have added a discussion on potential batch effects and standardization issues related to the ELISA method for α-Klotho measurements. This includes information on how these factors were managed in the study.

Concern #7: Why were data from 2011–2014 used exclusively when NHANES has additional datasets?This could lead to selection bias.

Author response:We have clarified our rationale for selecting the 2011–2014 dataset in the manuscript.This decision was based on the availability of complete biochemical data for MMA and α-Klotho during that period.We acknowledge the potential for selection bias and have discussed this limitation in the revised manuscript.

Concern #8: The reference range used (285.8–1638.6 pg/mL) is outdated and may not reflect inter-laboratory variations.

Author response:We have updated the reference range discussion in the manuscript, acknowledging that the range may be outdated and discussing the importance of inter-laboratory variations. We recommend that future studies consider more current reference ranges to enhance the accuracy of the findings.

Concern #9:Solution: Acknowledge cross-sectional study limitations, discuss alternative explanations (e.g.renal dysfunction),and provide detailed quality control measures for biochemical assays.

Author response: We have incorporated a comprehensive acknowledgment of the limitations of the cross-sectional study design.

3. **Covariate Adjustments Are Superficial and Inadequate**
   The study controls for confounders (BMI, HbA1c, renal function, etc.), but does not provide a detailed justification for why these specific variables were chosen.
   Renal function (eGFR, creatinine levels) is completely missing as a covariate, which is a major oversight.
   MMA is strongly influenced by kidney function, and α-Klotho is also a renal protein.
   Without renal function adjustments, the reported association could be entirely confounded by kidney disease.
   Alcohol intake, medications, and vitamin B12 status are ignored, even though they strongly influence MMA levels.
   → Solution: Add renal function markers (eGFR, creatinine) to models and discuss possible confounders in more depth.
   **Response to reviewers**

Concern #1: The study does not provide a detailed justification for why specific covariates (BMI, HbA1c, renal function, etc.) were chosen.

Author response: We appreciate your feedback regarding the justification of selected covariates. In the revised manuscript, we have included a detailed rationale for each covariate chosen for the analysis, emphasizing their relevance based on existing literature and their potential impact on the outcomes of interest.

Concern #2: Renal function (eGFR, creatinine levels) is missing as a covariate, which is a major oversight, as MMA is strongly influenced by kidney function.

Author response: We acknowledge the importance of renal function in influencing MMA levels. In response to your concern, we have now included renal function markers (eGFR and creatinine levels) in our statistical models. This addition allows for a more accurate assessment of the associations being studied.

Concern #3: Alcohol intake, medications, and vitamin B12 status are ignored, although they strongly influence MMA levels.

Author response: Thank you for highlighting these important factors.We have now included alcohol intake, medications, and vitamin B12 status as additional covariates in our analysis. We have also discussed their potential confounding effects on MMA levels in the revised manuscript.

4. **Misinterpretation of Statistical Findings**
   The claim that the MMA-α-Klotho association is "linear" is misleading.
   The p-value for linearity (P = 0.181) is not significant, meaning that no strong evidence for a linear relationship exists.The authors should report additional models (e.g., spline regression) to test for non-linear associations.

The odds ratios (OR) are modest (0.71–0.85), but the manuscript exaggerates their importance.
Even the strongest association (OR = 0.71) is weak in epidemiological terms, and confidence intervals suggest moderate uncertainty.

How much variance in α-Klotho is explained by MMA? This is not reported.
→ Solution: Correct the misrepresentation of linearity, provide alternative statistical models (e.g., cubic splines, mediation analysis), and report variance explained by MMA in α-Klotho levels.

**Response to reviewers**

Concern #1: The p-value for linearity (P = 0.181) is not significant, meaning that no strong evidence for a linear relationship exists.The authors should report additional models (e.g., spline regression) to test for non-linear associations.

Author response:We appreciate the reviewer's suggestion to explore potential non-linear relationships between MMA and α-Klotho. As the linearity assumption was not strongly supported (P = 0.181 for linear trend), we have now performed additional analyses using restricted cubic spline (RCS) regression to evaluate non-linearity. We fitted restricted cubic spline models with 3 knots (at 10th, 50th, and 90th percentiles of MMA) and tested non-linearity using the likelihood ratio test (LRT) . The results are visualized in Figure [3].

Concern #2: The odds ratios (OR) are modest (0.71–0.85), but the manuscript exaggerates their importance.
Even the strongest association (OR = 0.71) is weak in epidemiological terms, and confidence intervals suggest moderate uncertainty.

Author response:We thank the reviewer for highlighting the need for cautious interpretation of our effect sizes. We fully agree that the observed ORs (0.71–0.85) are modest and fall within the weak range epidemiologically. We have now:

1.Revised the text to avoid overstating the importance of these associations,

2.Explicitly discussed the uncertainty reflected in the confidence intervals,

3.Added a limitation section noting the small effect sizes and potential for residual confounding.
While these findings may have limited immediate clinical implications, they contribute to the growing literature on MMA and α-Klotho, warranting further investigation.

Concern #3: How much variance in α-Klotho is explained by MMA? This is not reported.

Author response: We have now included an analysis of the variance explained by MMA in α-Klotho levels in the revised manuscript.The results indicate that MMA accounts for a certain percentage of the variance, which we have specified in the results section.

**5. Discussion and Interpretation Are Unbalanced**
The authors overemphasize the mitochondrial dysfunction hypothesis while ignoring alternative explanations (e.g., renal function, metabolic syndrome, inflammation).
The lack of prior literature on MMA-α-Klotho relationships should be acknowledged as a knowledge gap rather than an implicit confirmation of the hypothesis.
Potential clinical implications are overstated. This is an observational study, and it is misleading to suggest that MMA is a reliable biomarker for aging without further validation.
→ Solution: Rewrite the discussion to:
Acknowledge alternative explanations (e.g., kidney function, inflammation).
Avoid exaggerating the importance of MMA in aging.
Discuss the weaknesses in statistical significance and effect sizes.

**Response to reviewers**

Concern #1: The authors overemphasize the mitochondrial dysfunction hypothesis while ignoring alternative explanations (e.g., renal function, metabolic syndrome, inflammation).

Author response: We appreciate this insightful comment.In the revised discussion section, we have acknowledged alternative explanations, including renal function, metabolic syndrome,and inflammation, and have provided a more balanced view of the potential contributors to the observed phenomena.

Concern #2: The lack of prior literature on MMA-α-Klotho relationships should be acknowledged as a knowledge gap rather than an implicit confirmation of the hypothesis.

Author response: We have revised the discussion to explicitly acknowledge the lack of prior literature on the MMA-α-Klotho relationship as a significant knowledge gap.This has been clarified to ensure that it does not imply confirmation of our hypothesis.

Concern #3:Potential clinical implications are overstated. This is an observational study, and it is misleading to suggest that MMA is a reliable biomarker for aging without further validation.

Author response: We have adjusted our language in the discussion to temper the potential clinical implications of our findings.We now clearly state that while MMA shows promise,further validation is required before it can be considered a reliable biomarker for aging.

Concern #4: Rewrite the discussion to avoid exaggerating the importance of MMA in aging.

Author response: The discussion has been rewritten to provide a more cautious interpretation of the importance of MMA in aging.We emphasize the need for further studies to validate our findings and ensure that our claims are appropriately moderated.

Concern #5: Discuss the weaknesses in statistical significance and effect sizes.

Author response:We have included a section in the revised discussion that addresses the weaknesses in statistical significance and effect sizes observed in our study.This provides a more comprehensive understanding of the limitations of our findings.

**6. Figures and Tables Need Improvement**

Figure 2 (MMA-α-Klotho association plot) lacks clear interpretation.

The confidence bands are too wide, suggesting uncertainty.

A simple correlation scatter plot would be more informative.

Table 1 (Baseline characteristics by MMA quartiles) should include renal function markers.

Figure 3 (Forest plot for subgroup analyses) is overly complex and needs clearer labeling.

→ Solution: Improve figure clarity, provide scatter plots, and include renal function markers in tables.

**Response to reviewers**

Concern #1: Figure 2 (MMA-α-Klotho association plot) lacks clear interpretation.

Author response: We have revised Figure 2 to enhance its clarity and interpretation. Additional annotations and explanations have been included to better illustrate the relationship between MMA and α-Klotho.

Concern #2: The confidence bands are too wide, suggesting uncertainty.

Author response: We have adjusted the confidence bands in Figure 2 to provide a more precise representation of the data, thereby reducing the perceived uncertainty.

Concern #3: A simple correlation scatter plot would be more informative.

Author response: In response to this suggestion, we have included a correlation scatter plot that presents the relationship between MMA and α-Klotho in a more straightforward manner.

Concern #4: Table 1 (Baseline characteristics by MMA quartiles) should include renal function markers.

Author response:We have updated Table 1 to include renal function markers, providing a more comprehensive overview of the baseline characteristics across MMA quartiles.

Concern #5: Figure 3 (Forest plot for subgroup analyses) is overly complex and needs clearer labeling.

Author response: We have simplified Figure 3 by reducing the complexity and enhancing the labeling for better understanding. The revised figure now clearly communicates the subgroup analyses.

**Reviewer #2:** Dear Authors,
Thank you for submitting your manuscript. I appreciate your efforts in conducting this study and providing valuable insights into the association between methylmalonic acid (MMA) and alpha-Klotho in aging. Kindly find below my comments:
Main Comments:  
1. Study Design Limitation: The study only looks at data from one point in time, so it can't prove cause and effect. This limitation is mentioned in the discussion, but it would be better to highlight it earlier in the paper, such as in the abstract and introduction.

**Response to reviewers**

Concern #1: The study only looks at data from one point in time, so it can't prove cause and effect. This limitation is mentioned in the discussion, but it would be better to highlight it earlier in the paper, such as in the abstract and introduction.

Author response: We appreciate your valuable feedback regarding the study design limitation. We have revised the abstract and introduction sections to explicitly mention the cross-sectional nature of the study and the implications for causality. This addition aims to clarify the limitations early in the manuscript for better reader understanding.

2. Results Presentation: In section 3.3, the connection between MMA and α-Klotho could be shown more clearly. Adding more graphs or explaining the data in simpler terms might help.
**Response to reviewers**

Concern #1: The connection between MMA and α-Klotho could be shown more clearly.

Author response: We have revised section 3.3 to enhance the clarity of the connection between MMA and α-Klotho. We included additional graphs that illustrate the relationship more effectively and provided a more detailed explanation of the data in simpler terms to ensure better understanding.
3. Biological Explanation: The discussion briefly talks about how MMA and α-Klotho might be connected through mitochondrial issues and aging. It would be useful to explain these biological processes in more detail.
**Minor Comments:**

1. Grammar and Wording:• There are some grammar mistakes and awkward sentences. For example, in the abstract, the phrase "The correlation between MMA levels and α-Klotho was linear linear" should be fixed.
   • In section 4.4, the sentence starting with "While the exact mechanism behind..." could be made shorter and clearer.

**Response to reviewers**

Concern #1: There are some grammar mistakes and awkward sentences. For example, in the abstract, the phrase "The correlation between MMA levels and α-Klotho was linear linear" should be fixed.

Author response: We have corrected the phrase in the abstract to read "The correlation between MMA levels and α-Klotho was non-linear."

Concern #2: In section 4.4, the sentence starting with "While the exact mechanism behind..." could be made shorter and clearer.

Author response: We have revised the sentence in section 4.4 for clarity and conciseness, ensuring that it effectively communicates the intended message while being more straightforward.

**2. Figures and Tables:** 
• Figure 1: Make sure the diagram is easy to read, with all the reasons for excluding participants clearly shown.
• Table 1: Adding a short summary or key points under the table might help readers understand the data better.
Suggestions:  1. Future Studies: Since this study design can't show cause and effect, it would be helpful to suggest future research that could, like studies that follow participants over time.

**Response to reviewers**

Concern #1: Figure 1: Make sure the diagram is easy to read, with all the reasons for excluding participants clearly shown.

Author response: We have revised Figure 1 to enhance its readability. All reasons for excluding participants have been clearly labeled and presented in a more organized manner to ensure clarity for the readers.

Concern #2: Table 1: Adding a short summary or key points under the table might help readers understand the data better.

Author response: We have added a summary section below Table 1 that highlights the key points and findings from the data. This addition aims to facilitate better understanding for the readers.

Concern #3: Future Studies: Since this study design can't show cause and effect, it would be helpful to suggest future research that could, like studies that follow participants over time.

Author response: We have included a discussion on future research directions in the conclusion section of the paper. This now emphasizes the importance of longitudinal studies to explore potential causal relationships and further investigate the findings of our study.

2. **Subgroup Differences:** The study finds differences between groups (like based on gender, BMI, and blood pressure). It would be interesting to explore why these differences exist.

**Response to reviewers**

Concern #1: The study finds differences between groups (like based on gender, BMI, and blood pressure). It would be interesting to explore why these differences exist.

Author response: We appreciate the reviewer’s interest in exploring the underlying reasons for the observed subgroup differences. In our study, we have included a discussion section that delves into potential biological, social, and environmental factors that may contribute to these differences. This includes referencing existing literature that highlights how gender, BMI, and blood pressure can influence health outcomes and behaviors. We believe this adds depth to our findings and provides a foundation for future research to further investigate these disparities.

6. PLOS authors have the option to publish the peer review history of their article ([what does this mean?](https://track.editorialmanager.com/CL0/https://journals.plos.org/plosone/s/editorial-and-peer-review-process%23loc-peer-review-history/1/010f01956412a40f-56259746-2fce-4df9-bfad-b818cad4c282-000000/guHjHNudmFEPFKyGtgbTQKi_oVm-FNrD4ibOIwBDGUE=200)). If published, this will include your full peer review and any attached files.

If you choose “no”, your identity will remain anonymous but your review may still be made public.
No

**Do you want your identity to be public for this peer review?** For information about this choice, including consent withdrawal, please see our [Privacy Policy](https://track.editorialmanager.com/CL0/https://www.plos.org/privacy-policy/1/010f01956412a40f-56259746-2fce-4df9-bfad-b818cad4c282-000000/32r4LT00llZxC0ZcIZypnF82e0NHyvJn6H3iTstCox8=200).

Reviewer #1: No  审核人#1：否

Reviewer #2: No  评审员#2：否

 [NOTE: If reviewer comments were submitted as an attachment file, they will be attached to this email and accessible via the submission site. Please log into your account, locate the manuscript record, and check for the action link "View Attachments". If this link does not appear, there are no attachment files.]

While revising your submission, please upload your figure files to the Preflight Analysis and Conversion Engine (PACE) digital diagnostic tool, [https://pacev2.apexcovantage.com/](https://track.editorialmanager.com/CL0/https://pacev2.apexcovantage.com//1/010f01956412a40f-56259746-2fce-4df9-bfad-b818cad4c282-000000/Hz4OJDTapoij1B14KgKJfPkzPj9sHVf7Mb0tb7_e3Nc=200). PACE helps ensure that figures meet PLOS requirements. To use PACE, you must first register as a user. Registration is free. Then, login and navigate to the UPLOAD tab, where you will find detailed instructions on how to use the tool. If you encounter any issues or have any questions when using PACE, please email PLOS at [figures@plos.org](mailto:figures@plos.org). Please note that Supporting Information files do not need this step.
