# Supplementary material for: Association between methylmalonic acid and Alpha-Klotho in American adults: A cross-sectional study
Source: PLoS One. 2025 Dec 30;20(12):e0337285. doi: 10.1371/journal.pone.0337285 (PMC12752970; doi:10.1371/journal.pone.0337285)
Supplement: Attachment — Submitted filename: REVIEW.docx [file pone.0337285.s005.docx]

Research Article Peer Review

Your research titled “Association Between Methylmalonic Acid and Alpha-Klotho in American Adults: Cross-Sectional Study” was reviewed and the following comments were made for your consideration.

**Abbreviations/Acronyms**

- When acronyms/abbreviations are stated in full, initial letters for such acronyms should be capitalized. Sentence 2, covariates section, “body mass index (BMI)” should be rectified as “Body Mass Index (BMI)”. This was similarly observed in sentence 3, covariate section, “Glomerular filtration rate (GFR)”. Please rectify these throughout the research protocol.

**Title**

- Please ensure consistency in the case of all proper nouns in the title. “methylmalonic”, “acid” and “adults” inconsistent with “Alpha-Klotho” in the title. Please ensure consistency of the case throughout the title.

**Abstract**

- Sentence 3 of materials and methods section, The author stated that “MMA is a continuous variable,” but in sentence 3 of the results section, the author categorized the MMA levels. Could the author please clarify.
- Sentence 1 of the conclusion, the author stated “significant association”. Could the author provide the corresponding p-value to corroborate the significance.
- Sentence 2 of the conclusion section, the author stated “mitochondrial dysfunction”, this makes the conclusion complex for comprehension, the author didn’t name or include any information about mitochondrial dysfunction through ought the abstract but only to include it at the conclusion. The author should please provide an information of how the “mitochondrial dysfunction” is connected to the study right from the beginning of the abstract but not include it at the conclusion without providing prior information and its linkages to the research.

**Materials and Methods**

- Sentence 1, materials and methods section, the author indicated “This cross- sectional study utilized NHANES data collected by Centers for Diseases Control and Prevention between **2011 and 2014**”. But sentence 1, measurement of serum alpha-klotho levels section, the author stated,” Serum alpha-klotho levels were analyzed in frozen samples from individuals aged 40-79 years, which were collected during NHANES from **2007 to 2016**, over the period from **2019 to 2020**”. The timelines stated are inconsistent or complex, could the author please clarify.
- Sentence 5, covariates section, could the author indicate or cite the source of the standards used in the BMI categorization. Could the author indicate the acceptable labels given to the categorizations. For instance, according to the World Health Organization (WHO), BMI< 18.5kg/m^2^ is underweight. Please provide more details.
- Similarly, smoking status, vitamin B12, among others. The author should please indicate the sources for the categorization including the standards. The author excellently showcased that with household income. The author should repeat that across all categorical variables.

**Results**

- The author should kindly report Standard deviations in parenthesis**.**

**General Comments**

- Even though, I was expecting the author to employ global acceptable tools for the assessment of certain variables in this study, For instance, Global Adult Tobacco Survey (GATS) for assessing participants smoking status, Global Physical Activity Questionnaire (GATS) to assess participants physical activity levels among others, nonetheless the author had done impressive work.
- Despite the author indicated the source of the data, much efforts should have been made to provide better detailed source of the data.
