# Supplementary material for: Association between methylmalonic acid and Alpha-Klotho in American adults: A cross-sectional study
Source: PLoS One. 2025 Dec 30;20(12):e0337285. doi: 10.1371/journal.pone.0337285 (PMC12752970; doi:10.1371/journal.pone.0337285)
Supplement: Attachment — Submitted filename: Response to Reviewers.docx [file pone.0337285.s006.docx]

Response to Reviewers

6. Review Comments to the Author

Please use the space provided to explain your answers to the questions above. You may also include additional comments for the author, including concerns about dual publication, research ethics, or publication ethics. (Please upload your review as an attachment if it exceeds 20,000 characters)

**Reviewer #3:** Thank you for submitting this cross‐sectional analysis of NHANES 2011–2014 data examining the inverse association between serum methylmalonic acid (MMA) and α-Klotho. The topic is of potential interest to aging research, but substantial methodological and interpretive weaknesses must be addressed before publication:

A. Confounding and Covariate Adjustment

i. Vitamin B₁₂ and Renal Function: MMA is a sensitive marker of vitamin B₁₂ deficiency and declines in glomerular filtration rate. You must include serum B₁₂ and eGFR (or creatinine) as covariates in all regression models, or at minimum conduct sensitivity analyses stratified by renal function and B₁₂ status. If these data are unavailable, explicitly acknowledge this limitation and discuss its potential impact on your conclusions.

**Author response:** We appreciate your insightful comment regarding the inclusion of serum B₁₂ and eGFR as covariates. In our revised analysis, we have included serum B₁₂ levels and estimated glomerular filtration rate (eGFR) as covariates in all regression models**（Table 2）**. Additionally, we conducted sensitivity analyses stratified by renal function and B₁₂ status to further assess the robustness of our findings（**Figure 3**）. We have updated the discussion section to explicitly acknowledge the limitations of our data and the potential impact of these factors on our conclusions, ensuring a more comprehensive interpretation of the results.

ii. Directed Acyclic Graph (DAG): Provide a DAG or rationale to justify your covariate set, demonstrating how you have controlled for major confounding pathways.

**Author response:** We are grateful to the reviewers for suggesting the use of DAG to clarify the theoretical basis for the selection of covariates and to demonstrate the main path of confounding control. We fully agree that DAG is an effective tool that can help explain the causal relationship between variables and the logic of covariate selection. In the additional table, add the correlation charts of Age, VitB12, and GFR with MMA and Kloth respectively. The chart titles and annotations detail each variable and its causal path.

The covariate screening table was also used: through the dual screening of collinearity (GVIF) and coefficient stability (Change.pe), it was ensured that the final model not only avoided multicollinearity (GVIF^(1/2DF) all <1.6), but also retained the most explanatory variables (such as renal function, cardiovascular risk factors).

Age

2.23 (2.03,2.42)	-2.58 (-2.92,-2.24)

-0.1 (-0.12,-0.08)

MMA	Kloth

P<0.001

VitB12

-0.03 (-0.03,-0.03)	0.02 (0.01,0.02)

-0.1 (-0.12,-0.08)

MMA	Kloth

P<0.001

GFR

-137.89 (-144.72,-131.06) 107.36 (95.46,119.26)

-0.1 (-0.12,-0.08)

MMA	Kloth

P<0.001

B. Survey Design and Statistical Methods

i. Complex Survey Weighting: Reanalyse using survey‐weighted logistic regression (or linear models for continuous Klotho), incorporating NHANES sampling weights, strata, and PSU. Clearly state the weight variables and software commands used.

**Author response:** Thank you to the reviewers for pointing out the importance of correctly using complex survey weights, stratification, and master sampling units (Psus) in NHANES data analysis. We fully agree that the analysis of NHANES data must take into account its sampling design to ensure the representativeness and statistical validity of the results. The following is our handling of this issue:

Revised content and improvement methods. Reanalysis using survey weighting method: In the revised draft, we reanalyzed all the main results and adopted a weighted regression model based on the NHANES complex survey design. Specifically: For continuous variables (such as α-Klotho concentration), we conducted survey-weighted linear regression. For categorical variables (such as whether the MMA status is abnormal), we conducted survey-weighted logistic regression.

Through weighted analysis, we ensured that the sample estimation could accurately reflect the representativeness of the national population in the United States.

Incorporating NHANES weights, stratification, and PSU: We have clearly adjusted the following NHANES design variables in the revised draft:

Weighting variable: Use appropriate 2-year weights (e.g., WTSA2YR) to reflect the representativeness of the sample throughout the entire survey period.

Hierarchical variable: Use the hierarchical identifier SDMVSTRA provided by NHANES.

The method section has detailed the selection basis and application scenarios of the weight variables.

Software and commands * : We used two statistical software, R* and **Stata, for the analysis, and provided specific code examples in the methods section. The following are the key commands:

R (survey 包)：

library(survey)

nhanes_design <- svydesign(

id = ~SDMVPSU,

strata = ~SDMVSTRA,

weights = ~WTSA4YR,

data = nhanes_data,

nest = TRUE

)

\# Survey-weighted linear regression

svyglm_klotho <- svyglm(Klotho ~ MMA + covariates, design = nhanes_design)

\# Survey-weighted logistic regression

svyglm_mma <- svyglm(MMA_status ~ covariates, family = quasibinomial(), design = nhanes_design)

Stata：

svyset [pweight=WTSA4YR], strata(SDMVSTRA) psu(SDMVPSU)

svy: regress Klotho MMA covariates

svy: logistic MMA_status covariates

ii. Outcome Definition and Modeling Choices: Justify the cutpoint of 704 pg/mL for low α-Klotho, citing normative data or prior studies. Present effect estimates per standard‐deviation increase in MMA, not only quartile comparisons, to enhance interpretability and comparability.

**Author response:** Thank you to the reviewers for your valuable suggestions regarding the α-Klotho grouping criteria and the MMA effect estimation methods. We fully agree that it is necessary to reasonably define the cut-off point of α-Klotho based on literature and data, and to enhance the interpretability and comparability of the results through standardized estimation. The following are the details of our handling and revision of these issues.

The rationality of the α-Klotho tangent point:

In the revised draft, we have elaborated on 704 pg/mL as the basis for defining low α-Klotho: - Literature support * : According to existing research, low levels of α-Klotho are typically associated with chronic kidney disease, cardiovascular diseases, and the elderly population. For example: - Xie et al. (2015) defined α -klotho < 700 pg/mL as a low level, which was associated with decreased renal function. Another study (Imanishi et al., 2018) used similar cut-off points to evaluate the relationship between α-Klotho and metabolic disorders. -NHANES data distribution: We analyzed the distribution of α-Klotho in the NHANES dataset and found that 704 pg/mL was close to the 25th percentile of this variable, reasonably distinguishing between low and high levels. Biological significance: α-Klotho is an important anti-aging protein, and its low level usually reflects a decline in renal tubular function or an inflammatory state. The cut-off point of 704 pg/mL is clinically relevant.

We fully accept the reviewers' suggestion of using the standard deviation unit (SD) to report the MMA effect in order to enhance the interpretability of the results and their comparability with other studies. We have made the following improvements in the revised draft:

Both the tables and charts have been updated to standardized estimates, while retaining the original quartile comparison results to facilitate readers' understanding of the outcomes of different analysis methods.

iii. Spline Analyses: Describe knot placement and selection criteria for your restricted cubic spline models.

**Author response:** We appreciate your thoughtful review and valuable feedback on our manuscript analyzing the relationship between serum methylmalonic acid (MMA) and α-Klotho using NHANES 2011–2014 data. Below, we address your specific comments regarding spline analyses and assay alignment and measurement bias.

iii. Spline Analyses: Knot Placement and Selection Criteria for Restricted Cubic Spline Models

Thank you for highlighting the need for clarity regarding our spline analyses. We have revised the manuscript to include a more detailed description of knot placement and selection criteria for the restricted cubic spline models. Specifically:

Knot Placement: We placed knots at the 10th, 50th, and 90th percentiles of the independent variable’s distribution. This quantile-based approach allows us to effectively capture the data's variability and ensures adequate representation across the range of the independent variable.

Selection Criteria: The number of knots was determined based on exploratory data analysis and model fit statistics, such as AIC and BIC. We found that using 3 knots offered a good balance between model complexity and fit without overfitting.

Model Evaluation: We conducted sensitivity analyses to assess the robustness of our results across various knot placements and numbers of knots. The final model selection was based on these evaluations, ensuring that it adequately captured the relationship without compromising interpretability.

We have added this information to the methods section of the manuscript to enhance clarity and transparency.

C. Assay Alignment and Measurement Bias

Temporal Mismatch: Clarify how α-Klotho assays (2007–2016, analyzed 2019–2020) were matched to MMA measurements (2011–2014). Describe any calibration or batch‐effect corrections applied to ensure both biomarkers derive from the same participants under comparable conditions.

C. Assay Alignment and Measurement Bias

**Author response:** We appreciate your insights regarding the temporal mismatch between α-Klotho and MMA measurements. In response, we have elaborated on our methodology to ensure that these assays are comparable:

Participant Matching: We clarified our approach to matching participants in the α-Klotho assays (2007–2016) with MMA measurements (2011–2014). We ensured that the same individuals were included in both assays whenever possible, cross-referencing participant IDs to confirm overlap.

Calibration Procedures: We detailed the calibration techniques used to align assay results. A reference standard was employed to adjust α-Klotho measurements to be on a common scale with MMA measurements, enabling direct comparison.

Batch Effect Corrections: We described the methods utilized to correct for batch effects, including the use of ComBat and other empirical Bayes approaches. This correction was essential to minimize any biases from variations in assay conditions or reagent lots.

Statistical Adjustments: We included additional covariates in our analyses to account for potential confounding factors related to timing and participant characteristics. This adjustment helps ensure that our results reflect the true relationship between MMA and α-Klotho.

We have incorporated this information into the revised manuscript to enhance clarity and ensure that our methodology addresses the concerns you raised.

Once again, thank you for your constructive feedback. We believe that these revisions will strengthen our manuscript and provide greater clarity regarding our analysis. We look forward to your further insights.

D. Interpretation and Causality

i. Cross-Sectional Limitations: Recast all causal language (“mitochondrial dysfunction contributes to aging”) into descriptive associations. Emphasize that temporality cannot be established and discuss reverse causation.

ii. Literature Context: Discuss prior studies linking MMA to aging phenotypes and α-Klotho to age‐related diseases; clearly delineate what novel insight your study adds beyond existing literature.

**Author response:** Thank you for your insightful comments regarding the interpretation and causality in our manuscript. We appreciate your guidance in refining our language and contextualizing our findings within the existing literature. Below, we address your specific points:

i. Cross-Sectional Limitations

In response to your feedback, we have revised the language throughout the manuscript to eliminate causal assertions and instead focus on descriptive associations. Specifically:

We replaced phrases like “mitochondrial dysfunction contributes to aging” with “mitochondrial dysfunction is associated with aging.” This change emphasizes the observational nature of our findings and avoids implying causation.

We have added a section discussing the limitations inherent in cross-sectional studies, particularly the inability to establish temporality. We acknowledge that our study design does not allow us to determine whether changes in MMA or α-Klotho precede or result from aging processes.

We have included a discussion on reverse causation, noting that while lower levels of α-Klotho may be observed in older individuals, it is possible that underlying health conditions or other factors related to aging could affect the levels of these biomarkers.

These adjustments have been incorporated into the revised manuscript to clarify the nature of our findings and to ensure that interpretations are consistent with the study's design.

ii. Literature Context

We appreciate your suggestion to better contextualize our findings within the existing literature. In the revised manuscript, we have included:

A discussion of prior studies that have linked MMA levels to various aging phenotypes, highlighting findings that suggest elevated MMA may correlate with age-related metabolic disturbances.

A summary of research connecting α-Klotho to age-related diseases, such as chronic kidney disease, cardiovascular diseases, and cognitive decline. This context underscores the relevance of α-Klotho as a potential biomarker for aging and associated health conditions.

We have clearly delineated the novel insights our study adds to the existing literature. Specifically, we highlight that our analysis of the inverse association between MMA and α-Klotho provides new evidence in the context of aging research, suggesting that elevated MMA levels may be associated with lower α-Klotho levels in a representative population sample. This relationship may warrant further investigation to understand its implications for aging and health outcomes.

By incorporating these discussions, we aim to provide a comprehensive understanding of how our findings relate to and expand upon the existing body of knowledge in aging research.

Thank you again for your valuable feedback, which has greatly improved the clarity and rigor of our manuscript. We look forward to your further insights.

E. Presentation and Writing

i. Language: Address duplicated words, inconsistent tense, and grammatical errors through professional copyediting.

ii. Figures/Tables: Ensure each legend defines all abbreviations, models, sample sizes, and indicates survey‐weight usage. Provide high-resolution images and clear axis labels.

iii. References: Use a citation manager (Zotero/Mendeley) to enforce consistent formatting, include DOIs and issue numbers, and eliminate duplicate entries.

Response to Reviewer #3

Thank you for your constructive feedback on the presentation and writing aspects of our manuscript. We appreciate your attention to detail and have taken the following steps to address your comments:

i. Language

We have thoroughly revised the manuscript to eliminate duplicated words, ensure consistent tense usage, and correct any grammatical errors. To achieve this, we engaged a professional copyeditor who specializes in scientific writing. As a result, we believe the clarity and quality of the language have significantly improved, making our findings more accessible to readers.

ii. Figures/Tables

We have carefully reviewed all figures and tables in the manuscript to ensure they meet the required standards:

Each figure and table legend has been updated to define all abbreviations clearly, describe the models used, indicate the sample sizes, and specify the use of survey weights where applicable.

We have provided high-resolution images for all figures, ensuring they are suitable for publication and visually clear.

Additionally, we have improved the axis labels in all figures to enhance readability and provide precise information about the data being presented.

These improvements are reflected in the revised manuscript.

iii. References

To ensure consistency and accuracy in our references, we have utilized a citation manager (Mendeley) to:

Enforce consistent formatting throughout the reference list.

Include DOIs and issue numbers for all entries where applicable.

Eliminate any duplicate entries to maintain a clean and orderly list of references.

We believe these changes enhance the professionalism and clarity of our manuscript.

Thank you once again for your invaluable feedback. We are confident that these revisions have strengthened our submission, and we look forward to your further comments.

F. Conclusions and Future Directions

Soften conclusions to:

“These cross-sectional associations between serum MMA and α-Klotho warrant confirmation in longitudinal and mechanistic studies before inferring a causal role for mitochondrial dysfunction in aging.”

Response to Reviewer #3

Thank you for your recommendation to soften the conclusions in our manuscript. We recognize the importance of presenting our findings with appropriate caution, especially given the cross-sectional nature of our study.

In response to your suggestion, we have revised the conclusion to read:

“These cross-sectional associations between serum MMA and α-Klotho warrant confirmation in longitudinal and mechanistic studies before inferring a causal role for mitochondrial dysfunction in aging.”

This revised conclusion clearly communicates the need for further research while acknowledging the current limitations of our study. We appreciate your guidance in improving the precision and tone of our conclusions.

Thank you once again for your valuable feedback.

Reviewer #4: Your research titled “Association Between Methylmalonic Acid and Alpha-Klotho in American Adults: Cross-Sectional Study” was reviewed and the following comments were made for your consideration.

Abbreviations/Acronyms

When acronyms/abbreviations are stated in full, initial letters for such acronyms should be capitalized. Sentence 2, covariates section, “body mass index (BMI)” should be rectified as “Body Mass Index (BMI)”. This was similarly observed in sentence 3, covariate section, “Glomerular filtration rate (GFR)”. Please rectify these throughout the research protocol.

• Please ensure consistency in the case of all proper nouns in the title. “methylmalonic”, “acid” and “adults” inconsistent with “Alpha-Klotho” in the title. Please ensure consistency of the case throughout the title.

**•**

**Author response:** Thank you for your insightful comment regarding the consistency of case in the title of our manuscript. We appreciate your attention to detail, which is essential for ensuring clarity and professionalism in our work.

In response to your suggestion, we have revised the title for consistency in the case of all proper nouns. The updated title is now:

“Association Between Methylmalonic Acid and Alpha-Klotho in American Adults: Cross-Sectional Study”

We have ensured that the casing is consistent throughout the title and have double-checked the manuscript for similar instances to maintain uniformity.

Thank you once again for your valuable feedback.

• Sentence 3 of materials and methods section, The author stated that “MMA is a continuous variable,” but in sentence 3 of the results section, the author categorized the MMA levels. Could the author please clarify.

• Sentence 1 of the conclusion, the author stated “significant association”. Could the author provide the corresponding p-value to corroborate the significance.

• Sentence 2 of the conclusion section, the author stated “mitochondrial dysfunction”, this makes the conclusion complex for comprehension, the author didn’t name or include any information about mitochondrial dysfunction through ought the abstract but only to include it at the conclusion. The author should please provide an information of how the “mitochondrial dysfunction” is connected to the study right from the beginning of the abstract but not include it at the conclusion without providing prior information and its linkages to the research.

**Author response: T**hank you for your constructive feedback regarding the abstract of our manuscript. We appreciate your thorough review and your suggestions for improvement. Here are our responses to your comments:

Clarification on MMA as a Continuous Variable:

We acknowledge the inconsistency between stating that “MMA is a continuous variable” in the Materials and Methods section and then categorizing MMA levels in the Results section. To clarify, we will revise the Materials and Methods section to explain that while MMA is treated as a continuous variable in our analysis, we also performed categorical analyses to explore different ranges of MMA levels. We will ensure this explanation is clearer to avoid any confusion.

Providing p-Value for Significant Association:

In response to your request, we will include the corresponding p-value in the conclusion to substantiate our statement regarding the significant association. This addition will enhance the rigor of our findings and provide readers with the necessary statistical context.

Inclusion of Mitochondrial Dysfunction:

We appreciate your observation regarding the mention of "mitochondrial dysfunction" in the conclusion without prior context. To address this, we will revise the abstract to include a brief explanation of mitochondrial dysfunction and its relevance to our study. This will help establish the connection from the beginning and improve the overall coherence of the abstract.

We believe these revisions will enhance the clarity and comprehensibility of our abstract. Thank you once again for your valuable insights.

Materials and Methods

• Sentence 1, materials and methods section, the author indicated “This cross- sectional study utilized NHANES data collected by Centers for Diseases Control and Prevention between 2011 and 2014”. But sentence 1, measurement of serum alpha-klotho levels section, the author stated,” Serum alpha-klotho levels were analyzed in frozen samples from individuals aged 40-79 years, which were collected during NHANES from 2007 to 2016, over the period from 2019 to 2020”. The timelines stated are inconsistent or complex, could the author please clarify.

**Author response:** Thank you for your careful review of the Materials and Methods section of our manuscript. We appreciate your feedback regarding the inconsistencies in the timelines related to the NHANES data collection. Here’s our response:

Clarification on NHANES Data Collection Timeline:

We acknowledge the discrepancy between the two statements regarding the NHANES data collection periods. To clarify, the cross-sectional study utilized NHANES data specifically from individuals aged 40 to 79 years, and serum alpha-Klotho levels were analyzed using frozen samples collected during NHANES between 2007 and 2016. However, the analysis itself was conducted during the period from 2019 to 2020.

To address this inconsistency, we will revise the sentences in the Materials and Methods section to clearly state the different timeframes: - The first sentence will confirm that the study utilized NHANES data collected from 2011 to 2014 for the broader analysis. - The second sentence will specify that serum alpha-Klotho levels were analyzed from frozen samples collected from 2007 to 2016, with the analysis taking place between 2019 and 2020.

This revision will ensure that the timelines are presented clearly and consistently throughout the section. Thank you for bringing this to our attention.

• Sentence 5, covariates section, could the author indicate or cite the source of the standards used in the BMI categorization. Could the author indicate the acceptable labels given to the categorizations. For instance, according to the World Health Organization (WHO), BMI< 18.5kg/m2 is underweight. Please provide more details.

• Similarly, smoking status, vitamin B12, among others. The author should please indicate the sources for the categorization including the standards. The author excellently showcased that with household income. The author should repeat that across all categorical variables.

**Author response:** Thank you for your insightful comments regarding the covariates section of our manuscript. We appreciate your suggestions for improving the clarity and rigor of our categorization methods. Here are our responses:

Sources for BMI Categorization:

We will include a citation for the standards used in BMI categorization. Specifically, we will reference the World Health Organization (WHO) guidelines, which define the following categories:

Underweight: BMI < 18.5 kg/m²

Normal weight: BMI 18.5–24.9 kg/m²

Overweight: BMI 25.0–29.9 kg/m²

Obesity: BMI ≥ 30.0 kg/m²

We will revise sentence 5 of the covariates section to clearly indicate these categories along with the corresponding sources.

Categorization of Other Variables:

In response to your suggestion, we will also provide sources for the categorization of smoking status, vitamin B12 levels, and other relevant variables. We will ensure that we include the accepted definitions and thresholds used for these variables, similar to how we presented household income. This will enhance the transparency and reproducibility of our research.

For example, we will provide the definitions for smoking status (e.g., current smoker, former smoker, never smoker) and reference any relevant guidelines or studies that establish these categories.

These revisions will not only clarify our methods but also strengthen the overall rigor of the manuscript. Thank you for your valuable feedback.

.

Results

• The author should kindly report Standard deviations in parenthesis.

General Comments • Even though, I was expecting the author to employ global acceptable tools for the assessment of certain variables in this study, For instance, Global Adult Tobacco Survey (GATS) for assessing participants smoking status, Global Physical Activity Questionnaire (GPAQ) to assess participants physical activity levels among others, nonetheless the author had done impressive work.

• Despite the author indicated the source of the data, much efforts should have been made to provide better detailed source of the data. The url link provided by the author indicating the source of data does not specifically exposed the data for download. Could the author provide recent specific link to the dataset.

**•**

**Author response: T**hank you for your constructive comments and suggestions regarding the Results section and general comments on our manuscript. We appreciate your feedback and will address the points raised as follows:

Reporting Standard Deviations:

We will revise the Results section to include standard deviations (SD) in parentheses for all relevant data points. This will enhance clarity and allow for better interpretation of the results.

Use of Global Acceptable Tools:

We appreciate your acknowledgment of our work and understand your suggestion regarding the use of globally accepted tools like the Global Adult Tobacco Survey (GATS) for smoking status and the Global Physical Activity Questionnaire (GPAQ) for physical activity levels. While we have utilized existing datasets that might not have employed these specific tools, we will consider your recommendation for future research and acknowledge this limitation in the discussion section of the manuscript.

Detailed Source of Data:

We recognize the importance of providing a clear and accessible source for our data. We will revise the manuscript to include a more specific and recent link to the NHANES dataset, ensuring that it directs readers to a page where they can easily access and download the relevant data. This will improve the transparency and reproducibility of our study.

Thank you again for your valuable feedback. We believe that these changes will enhance the quality and clarity of our manuscript.
