# Supplementary material for: Association between methylmalonic acid and Alpha-Klotho in American adults: A cross-sectional study
Source: PLoS One. 2025 Dec 30;20(12):e0337285. doi: 10.1371/journal.pone.0337285 (PMC12752970; doi:10.1371/journal.pone.0337285)
Supplement: Attachment — Submitted filename: Response to Reviewer10.16.docx [file pone.0337285.s007.docx]

**Response to Reviewer10.16**

We note that there were multiple versions of Manuscript in your submission's file inventory. We have removed the older file(s), retaining the most recent version(s) for editorial review. Please double check your submission file inventory and let us know if any files appear to be outdated or absent.

**Reply：**

Thank you for tidying up the file inventory and for your prompt notification.

We have reviewed the remaining files in the submission system and confirm that all current, up-to-date documents are present; no essential items are missing or obsolete. The versions now retained are the ones we intend to be considered for editorial review.
